# Stay or go? Changing breeding conditions affect sexual difference in colony attendance strategies of Atlantic puffins *Fratercula arctica*

**DOI:** 10.1002/ece3.11681

**Published:** 2024-07-10

**Authors:** Tycho Anker‐Nilssen, Martina Kadin, Christoffer Høyvik Hilde

**Affiliations:** ^1^ Norwegian Institute for Nature Research Trondheim Norway; ^2^ Swedish Museum of Natural History Stockholm Sweden; ^3^ School of Aquatic & Fishery Sciences University of Washington Seattle Washington USA; ^4^ Norwegian Institute for Nature Research Bergen Norway

**Keywords:** breeding conditions, colony attendance, *Fratercula arctica*, life history trade‐offs, sex‐ and age‐specific responses

## Abstract

Male and female birds have different roles in reproduction and, thereby in their reproductive investment, which in turn may increase negative effects of poorer breeding conditions caused by e.g., climate change or ecosystem regime shifts. By using a 33‐year time series of resightings of Atlantic puffins *Fratercula arctica* individually colour‐ringed as breeders in previous years, we showed that the difference in colony attendance of male and female birds depended on the environmental conditions for raising young, proxied by the average duration of the chick period and size of the herring *Clupea harengus* fed to the chicks in the colony each year. The longer the chick period, the more was the sex ratio of adults sitting visibly in the colony biased in favour of males. An increase in herring size, indicating better feeding conditions for raising chicks, led to more observations of both sexes. Additionally, we found that birds were observed less with age and females more so than males. We discuss the results in relation to general life‐history theory on sexual differences in trade‐offs between individual investment in breeding and own survival. Our results suggest that females are increasingly more willing than males to invest in provisioning for the chick the more and longer the chick needs such care.

## Graphical abstract

Using a 33‐year CMR data set, we show that colony attendance of the Atlantic puffin differs between the sexes and in their response to environmental conditions for raising young, as proxied by the duration of chick rearing and the quality of their main prey. Our results suggest females are increasingly more willing than males to invest in provisioning for the chick the longer the chick needs such care. We discuss the results in relation to general life‐history theory on sexual differences in trade‐offs between individual investment in breeding and own survival.

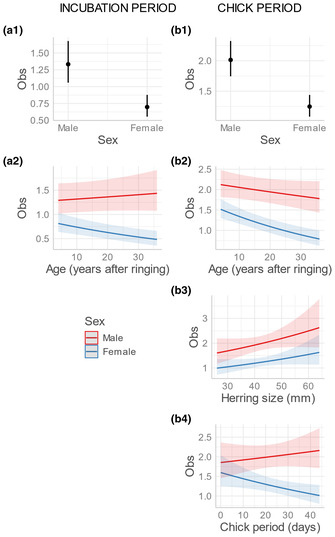

## 1 INTRODUCTION

Many marine vertebrate populations have a slow life history characterised by a high adult survival rate and low annual productivity (Stearns, 1992). In fluctuating environments with highly variable breeding conditions, adults of long‐lived species need to balance their investments in reproduction without jeopardising their chances of survival to the next breeding season (Drent & Daan, 1980; Williams, 1966). The individuals within a population of long‐lived species will, however differ in terms of what will be the optimal level of reproductive investment to maximise their lifetime reproductive success, which at any time is likely to vary with factors such as their age, previous success, physical condition, food availability, predation risks and how much they and their partners, have already invested in the current breeding attempt (Stearns, 1992). Given inherent sexual differences in physiological costs to produce offspring, males and females are likely to differ in their efforts to protect and sustain the clutch (Clutton‐Brock, 2017; Sanz‐Aguilar et al., 2012; Tavecchia et al., 2001). Such differences could act to increase negative effects of poorer conditions for breeding, which may often be an expected result of climate change or regime shifts in marine ecosystems, as demonstrated for guillemots of genus *Uria* and kittiwakes *Rissa tridactyla* (Descamps et al., 2017; Irons et al., 2008) and by the general inability among 61 species of seabird to adjust their timing of breeding to buffer increasing sea temperatures (Keogan et al., 2018). To what extent the sexes' willingness to invest in their offspring is differently affected by fluctuations in environmental conditions has, however rarely been quantified, although sexual differences in the probability of skipping reproduction, which also were related to parallel differences in survival, have been documented in procellariform seabirds (e.g., Cruz‐Flores et al., 2021).

The Atlantic puffin *Fratercula arctica* (hereafter puffin) is a medium‐sized burrow‐nesting auk (Alcidae) that breeds on most boreal and low‐arctic coasts of the North Atlantic (e.g., Nettleship & Birkhead, 1985). Puffins are highly pelagic and only come ashore to breed, but their breeding season is usually 3–4 months long, with incubation of the single egg taking about 42 days (Myrberget, 1962) and the typical nestling period being almost equally long (Harris & Wanless, 2011). They are long‐lived (Harris et al., 2005) and monogamous (Anker‐Nilssen et al., 2008) and exhibit strong nest‐site fidelity, only occasionally moving to a neighbouring nest burrow when losing their partner (Harris & Wanless, 2011; Wernham, 1993). Although the sexes are not very different in size, with males weighing only 8–10% more than females during chick rearing (Anker‐Nilssen et al., 2008), male puffins invest more in territorial defence, while the females invest more in direct care (incubation and chick‐provisioning) of the offspring (Creelman & Storey, 1991). A sexual difference in incubation effort is, however not confirmed by other studies (summarised by Harris, 1986), but most of them suffer from small data sets and some results could also be biased by males and females not reacting in the same way to various registration methods inside the nest burrow (Ashcroft, 1976). Nevertheless, even if balanced with differences in other activities, we hypothesise that the initial difference in reproductive investment between males and females in terms of egg production alone makes it likely that their investments in other reproductive tasks are affected differently by changes in environmental conditions for breeding. As shown by Landsem et al. (2023), there is a slight tendency for female puffins to have lower survival than males and although they found no evidence of sexual differences in actuarial senescence of adults, a higher rate of senescence in colonies producing well indicate puffins do invest more in parental care when conditions for breeding are favourable.

In this study, we test these hypotheses by using a 33‐year time series on resightings of individually colour‐ringed adult puffins breeding on Røst in the Norwegian Sea to explore if colony attendance of male and female birds, as a proxy of their investment in parental care, is affected by environmental conditions during the breeding season. Annual monitoring of key demographic parameters and chick diet of the study population was initiated in the 1960s and it has since been subject to a wide range of ecological research, too extensive to summarise here. A key finding of several studies is however the Røst puffins' strong dependence on herring *Clupea harengus* to breed successfully (e.g., Anker‐Nilssen, 1992; Cury et al., 2011; Durant et al., 2003). These analyses demonstrate that the fledging success of puffin chicks in this colony for decades has been relying on both the abundance and quality of first‐year (age 0) herring produced by the Norwegian spring‐spawning stock, which drift past the colony in summer on their way to their nursery areas in the Barents Sea (Anker‐Nilssen, 1992; Cury et al., 2011; Durant et al., 2003). The huge inter‐annual changes in the availability of age 0 herring within reach of the colony is the main explanation for why this puffin population on average has experienced high breeding success in only one of three seasons over the 60‐year period 1964–2023, with near‐total or complete breeding failures at the population level in most years (Anker‐Nilssen, unpubl. data; Cury et al., 2011; Durant et al., 2003). The larger this herring was in the chick diet, the higher success; when fed only transparent herring larvae, most chicks die prematurely, whereas most survive to fledging when fed larger, smoltified herring (the time herring also start aggregating in schools). The average length of the chick period (i.e., from hatching to either death or nest departure), is even more strongly correlated with sea temperature and salinity (Durant et al., 2006), key indices of the influx of Atlantic water masses and processes important for the production of suitable prey for chick‐feeding puffins on the Norwegian continental shelf (Sætre et al., 2002). We, therefore, used the average length of the chick period and the size of age 0 herring in their diet as proxies for the environmental conditions in each year.

On this background, we made the following predictions: (1) *Different roles*: males are observed more frequently than females because they spend more time out in the open on the colony to defend the nest, while females engage more in the direct care of the offspring (incubation, brooding, feeding), (2) *Stay or go*: both sexes are observed less frequently in the poorer years because they should invest more time searching for food and less time at the colony when conditions deteriorate and leave the colony when the conditions are no longer sufficient to sustain successful breeding and/or their own body condition, (3) *Unequal levels of investment*: the worse the breeding conditions get females are observed increasingly less often on the colony than males, as the female from the start has invested more energy in the offspring and therefore has more to lose by reducing her parental efforts and, (4) *Accumulated level of investment*: as the total amount of reproductive investment increases with time and energy spent in undertaking the different tasks, the difference between male and female colony attendance is also likely to increase the more and longer the parent birds invest in chick rearing, both within a season and, over their lifetime, with increasing age.

## 2 MATERIALS AND METHODS

### 2.1 Study area and affiliation

**FIGURE 1:**
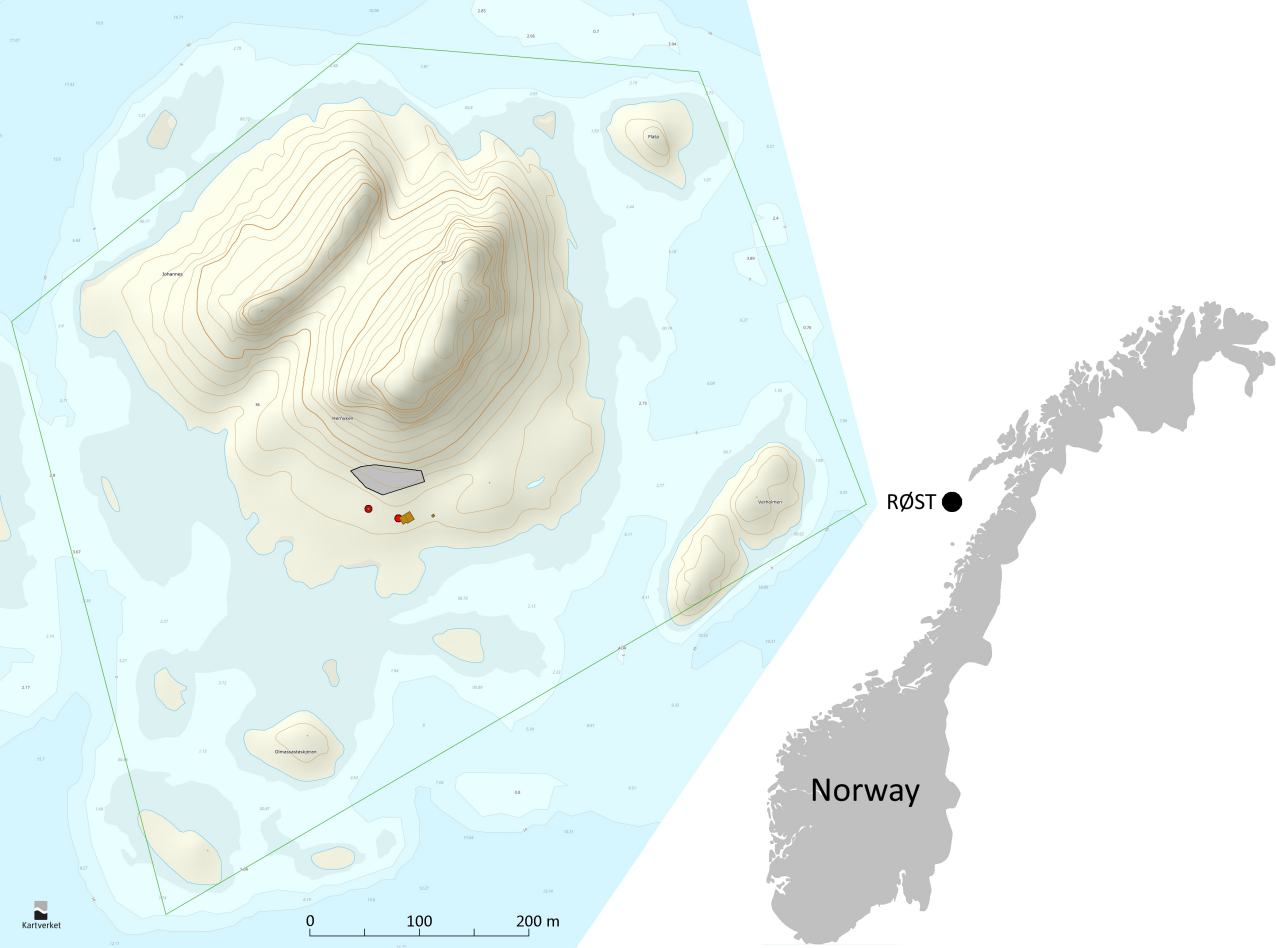
Topographic map of the island Hernyken in the Røst archipelago, with the nature reserve border (green line), study plot (grey area), observation stands (red circles) and field station (brown building) indicated. The equidistance is 5 m. (*Map source*: © Kartverket, www.norgeskart.no).

All field data for this study (Sections 2.2, 2.5) were collected on a fixed study plot (Figure 1) as part of the annual monitoring of the demographic performance of the puffin population on the island of Hernyken (67°26′N, 11°52′ E) in the Røst archipelago, northern Norway. The monitoring was initiated with breeding success parameters already in 1964, expanded with data on population trends and chick diet from 1979 and adult survival from 1990 and has since 2005 been maintained as an integrated part of the SEAPOP network of key‐site monitoring (www.seapop.no/en).

### 2.2 CMR data

We used the 33‐year capture‐mark‐recapture (CMR) data set collected at Hernyken for monitoring of adult puffin survival rate. All birds were captured by erecting every year one to three mist‐nets close to each other in the same spot within the study plot, covering together about 1000 m^2^ of a grassy slope with scattered big rocks (Figure 1), the typical breeding habitat of puffins on Hernyken (Anker‐Nilssen & Røstad, 1993). Between 1990 and 2021, a total of 577 adult puffins breeding in this area were mist‐netted and ringed with a numbered ring of stainless steel (if not already carrying one) and either an individually unique combination of two‐three Darvic colour rings (1990–1998, *n* = 247) or an individually coded yellow acrylic ring (1997–2019, *n* = 330). To make sure the birds were breeders, only those captured when bringing fish for their young were colour‐ringed. Because of very low feeding activity in nine consecutive years (2007–2015) when the study population experienced virtually complete breeding failure every year, exceptions to this rule were made in 2013 and 2014 to maintain an adequate sample size for monitoring of survival rates. This was justified by the total breeding failures at the population level at the study colony in nine consecutive years from 2007 onwards with no signs of non‐natal recruitment, implying that all adults present in the colony in those years were likely to be at least 7–8 years old, i.e., having reached the normal age of first breeding for the species (median of 7 years; Harris & Wanless, 2011).

In each subsequent year (1991–2022), visual searches for marked birds in the study plot and its immediate surroundings were, for efficiency reasons, made when reasonable numbers of birds were sitting out. Puffins are very social birds and synchronise their attendance at the colony, most often in cycles with 4–7 days between the peaks (e.g., Harris & Wanless, 2011). This happens throughout the breeding season with huge day‐to‐day variation, from virtually none to thousands of birds swamping the colony area. What triggers this behaviour is not known in much detail, but at Røst the peaks appear more irregular than in many other colonies and with much longer intervals in the seasons with the poorest breeding success (Anker‐Nilssen & Aarvak, 2006). In the study colony, there is continuous daylight for 6 weeks in mid‐summer and peak numbers usually build up in the evening, when the risk of predation from local great black‐backed gulls *Larus marinus* is lower than in the daytime (pers. observations).

The resighting work started in early May (start of egg laying) and continued from late incubation (early June) and throughout the breeding season. Birds spotted several times within an observation bout were only counted as one observation in the analysis. The annual observation effort ranged from 4.45 to 80.8 (mean 38.1) hours of effort spread on 7–65 (mean 32.9) observation bouts using a 40–70 × telescope from one of two fixed positions located within 20–60 m of all parts of the plot (Figure 1). Despite daily studies in other parts of the same colony, no colour‐ringed birds were ever spotted alive more than 20 m outside of the study plot.

### 2.3 Productivity data

Each year, a selection of 34–284 (mean 103) nest burrows containing an egg or small chick in early June were individually marked and checked regularly by hand at intervals of typically 3–5 (range 2–7) days until breeding success could be determined. Nest‐specific dates of hatching and chick death or fledging were later calculated from the burrow contents registered on the nest visits, most often guided by comparing measurements of chick size and growth rate (body mass and lengths of culmen, head + bill, tarsus and wing chord) with growth curves for chicks of known age and energy consumption (see Anker‐Nilssen & Aarvak, 2006; Øyan & Anker‐Nilssen, 1996).

### 2.4 Herring size

The prey in food loads carried by chick‐feeding puffins were sampled regularly each year (mean 75, range 0–266 loads), most often by mist‐netting birds in an area adjacent to where the CMR study was done and collecting the loads dropped under the net. All prey items were identified to species and their total length and fresh body mass were measured individually in the field to the nearest 1 mm and 0.01 g, respectively.

### 2.5 Sexing and ageing of birds

319 (55%) of the colour‐ringed birds were sexed by molecular DNA analysis based on a small blood sample (≈0.1 μL), following the protocol described by Anker‐Nilssen et al. (2017). The other birds were sexed from biometric measurements by applying a colony‐specific discriminant function based on the head + bill length and bill depth at gonys derived from 92 adults sexed by gonadal inspection (Anker‐Nilssen & Brøseth, 1998).

Since all but 13 birds were first ringed as adults, their true age was usually unknown. We, therefore, used time elapsed since first captured as an adult as a proxy for their age, an approach proven useful for studies of senescence in both common guillemots (Crespin et al., 2006) and puffins (Landsem et al., 2023). As the ringing of puffins with metal rings in the study plot started several decades earlier and colour ringing required proofs of breeding, 314 (54%) of the birds had already been captured as an adult in a year prior to when they got the colour ring(s) (on average 7.6 years earlier, range 1–23 years).

### 2.6 Data treatment

Birds not observed during a breeding season but known to be alive based on sightings in subsequent years were included in the analysis (with zero observations for the year in question). Birds were removed from the analysis after the last year they were re‐sighted.

The difference between the mean date of hatching and the mean date of chick death or nest departure was used as the average duration of the chick period in each year, hereafter referred to as the chick‐rearing period. In 4 years, when total or near‐total hatching failure at the population level made calculation of a mean hatching date impossible (no data) or very unreliable (*n* < 4 nests), we used 24 June as the mean hatching date (the average of all other study year means).

The annual average size of individual age 0 herring (mean *n* = 532, range 0–2191 fish) in the food loads collected from chick‐feeding adults was calculated by linear regression for 1 July each year, giving equal weight to the average for each five days covered by sampling. A strong linear relationship (28 years since 1980: *R* ^2^ = .548) between these herring size estimates and the ICES (International Council for the Exploration of the Sea) recruitment indices for age 2 herring 2 years later (ICES, 2023) was used to produce estimates of age 0 herring length for the 11 years when too few herring were sampled in the colony (1995, 2007–2014 and 2020–2021).

### 2.7 Statistical analyses

To test if sex distribution deviated from equality, we used an Excel® spreadsheet to apply a χ^2^‐test as described by Zar (1984) and corrected for continuity as described by Yates (1934). All other data analyses were performed using the software R (R Core Team, 2023).

#### 2.7.1 Modelling framework

To explore how our response variable, the annual frequency of observations of each bird within each period (i.e. resighting frequency), was affected by sex and environmental covariates, we applied a Poisson generalised linear mixed model with a log link function (Equation 1), using package *glmmTMB* (Brooks et al., 2017). As we hypothesised that the sexual difference in resighting rate, reflecting colony attendance, should be most apparent after hatching, separate analyses were done for observations made in the incubation period (i.e., from 42 days before the yearly mean hatching date, Myrberget, 1962) and those made in the subsequent chick‐rearing period.

#### 2.7.2 Fixed effects

Models were run with the following covariates: sex, age, herring size and observation effort. A dummy variable indicating whether a bird had been observed previously in the season (before the incubation or chick‐rearing period, respectively) was included in the models. In addition, the length of the chick period in days was included as a covariate for the chick‐rearing period analysis. We also tested for interactions between sex and three of the other covariates to investigate if there were any differences in how males and females responded to environmental cues (length of chick period and herring size) or their age when attending the colony.

#### 2.7.3 Random effects and offset variable

Bird ID (unique number on foot metal ring) was included as a random intercept effect to account for individual variability due to multiple observations per individual. Year was included as a random intercept effect to account for among‐year variation in observations due to non‐observed environmental factors. Observation effort was accounted for by including the log of the annual total time (in hours) spent observing as an offset variable.

(1) $$\begin{array}{c} {\mathrm{obs}}_{i,t}∼\text{Poisson}\left(\mu_{i,t}\right)\\ \begin{array}{c} E\left({\mathrm{obs}}_{i,t}\right)=\mu_{i,t}\\ \begin{array}{c} \log\left({\mathrm{obs}}_{i,t}\right)={\mathrm{sex}}_{i}+{\mathrm{age}}_{i}+\text{herring}\_{\text{size}}_{t}+\text{chick}\_{\text{period}}_{t}+{\mathrm{sex}}_{i}\times {\mathrm{age}}_{i}+{\mathrm{sex}}_{i}\\ \times \text{herring}\_{\text{size}}_{t}+{\mathrm{sex}}_{i}\times \text{chick}\_{\text{period}}_{t}+\text{seen}\_\text{befor}e_{i}+{\text{birdID}}_{i}\\ \begin{array}{c} +{\text{year}}_{t}+\left(\log\left({\text{effort}}_{t}\right)\right)\\ \begin{array}{c} {\text{birdID}}_{i}∼N\left(0,\delta^{2}\right)\\ {\text{year}}_{t}∼N\left(0,\delta^{2}\right) \end{array} \end{array} \end{array} \end{array} \end{array}$$

where obs_*i,t*_ is the number of observations of individual *i* in year *t*, sex_*i*_ is the sex of individual *i*, age_*i*_ is the age after first ringing of individual *i*, herring_size_*t*_ is the length of age 0 herring in year *t*, chick_period_*t*_ is the length of the chick‐rearing period in year *t* (not included in incubation period model), birdID_*i*_ and year_*t*_ are random intercepts assumed to be normally distributed with mean 0 and variance $\delta^{2}$. Effort_*t*_ is the number of hours spent observing in year *t*. *Model selection*: We selected the most parsimonious model based on the Akaike information criterion (AIC, Burnham & Anderson, 2002). Model diagnostics were done using the R‐package *DHARMa* (Hartig, 2022). The QQ‐plot and residuals vs. fitted values showed no major deviations from the model assumptions. An outlier test showed no significant deviations from the expected number of individual observations per year given the sample size during incubation (*p* = .20) but a significant deviation for the chick‐rearing period (*p* = .05). Removing the outliers had no effect on the estimates and they were therefore included in the model.

## 3 RESULTS

The sex distribution of the 577 colour‐ringed birds was heavily skewed in favour of females (340:237, χ^2^ = 18.03, df = 1, *p* < .001). Only 54 (9%) individuals were never re‐sighted in the field, whereas 18,062 resightings were made of the other 523 colour‐ringed adults in subsequent years with an average (mean ± SE) of 4.06 ± 0.06 observations per bird each year they were recorded.

**FIGURE 2:**
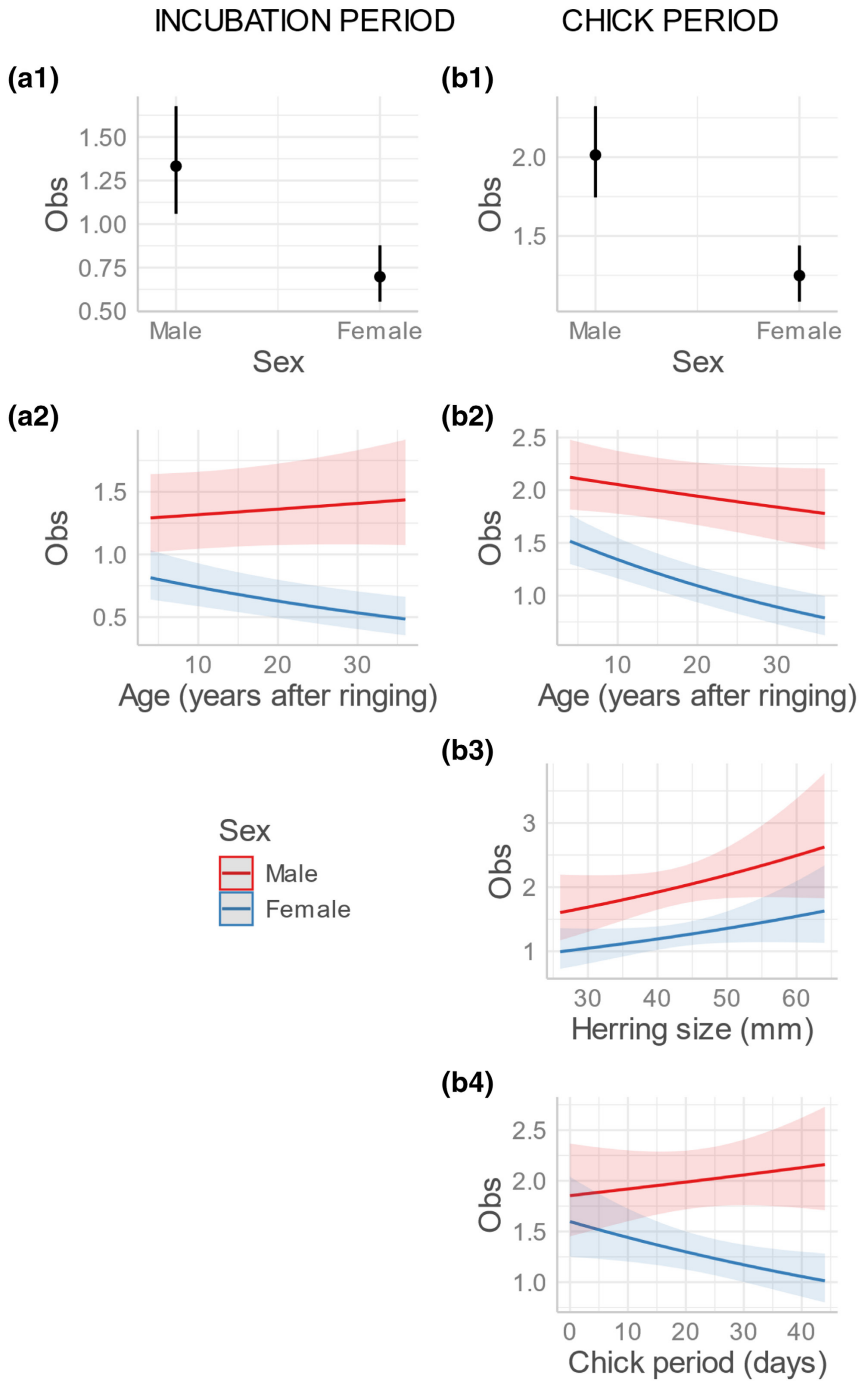
Estimated differences in numbers of observations (Obs) between male and female Atlantic puffins during the incubation period (panels on left) and chick‐rearing period (panels on right) in relation to sex (a1, b1), age in years after first ringed (a2, b2), herring size (total length) in the chick diet in mm (b3) and length of the chick period in days (b4). The trend lines with 95% confidence intervals for each predictor are indicated for mean values of the remaining predictors.

The best model for the likelihood of observing an individual in the incubation period included sex in interaction with age as an additive effect (Tables 1 and 2), whereas the preferred model for the chick‐rearing period included sex in interaction with age and length of chick period and herring size as an additive effect (Tables 1 and 3). As expected, male puffins were observed more often than females during both the incubation and chick period (estimated average no. of observations per incubation period: males 1.33 (CI: 1.06–1.68), females 0.70 (CI: 0.55–0.88); estimated average no. of observations per chick rearing period: males 2.01 (CI: 1.74–2.32), females 1.25 (CI: 1.08–1.44); Figure 2a1,b1). Resighting rate decreased more with age in females than in males during both incubation (slope_female_ = −0.016, slope_male_ = 0.004) and chick rearing (slope_female_ = −0.020, slope_male_ = −0.005) (Figure 2a2,b2). An increase in herring size led to a parallel increase in resighting frequency during chick rearing for both sexes (slope = 0.13) (Figure 2b3). The length of the chick period (in days) had a negative effect on resighting rate in females while the opposite was observed in males (slope_female_ = −0.010, slope_male_ = 0.004) (Figure 2b4).

**TABLE 1:** Effect of sex (where female is the baseline/intercept and sexM is the difference between males and females), age after first ringing, herring size and length of chick‐rearing period on the number of observations of individual Atlantic puffins *Fratercula arctica* on Hernyken, North Norway, 1991–2022 during incubation and chick rearing.

	Incubation			Chick rearing		
Covariate	Beta	*SE*	*p*	Beta	*SE*	*p*
Intercept (SexF)	−2.699	0.128	<.001	−2.657	0.332	<.001
sexM	0.383	0.083	<.001	−0.053	0.091	.559
chick_period				−0.010	0.004	.021
Age	−0.016	0.004	<.001	−0.020	0.004	<.001
herring_size				0.013	0.008	<.001
seen_before	0.261	0.041	<.001	0.278	0.031	<.001
sexM × chick_period				0.014	0.002	<.001
sexM × Age	0.020	0.006	<.001	0.015	0.005	.002

*Note*: Results are based on a generalised linear mixed model with bird ID and year as random effects.

**TABLE 2:** Model selection table ranging the top 10 models assessed for estimating numbers of observations per individual per year in the incubation period.

AIC	ΔAIC	Model
**10761.61**	**0**	**sex + age + seen_before + (1 | birdID) + (1 | year) + sex:age**
10763.17	1.56	sex × (age + herring_size) + seen_before + (1 | birdID) + (1 | year)
10763.52	1.91	sex + age + seen_before + herring_size + (1 | birdID) + (1 | year) + sex:age
10770.90	9.29	sex + herring_size + seen_before + age + (1 | birdID) + (1 | year) + sex:herring_size
10771.73	10.12	sex + herring_size + seen_before + (1 | birdID) + (1 | year) + sex:herring_size
10771.91	10.29	sex + age + seen_before + (1 | birdID) + (1 | year)
10772.67	11.06	sex + seen_before + (1 | birdID) + (1 | year)
10774.54	12.93	sex + herring_size + seen_before + (1 | birdID) + (1 | year)
10774.54	12.93	age + seen_before + (1 | birdID) + (1 | year)
10898.49	136.88	age + herring_size + seen_before + (1 | birdID) + (1 | year)

*Note*: Preferred model marked in bold. All models have observation effort (hours) as an offset term.

**TABLE 3:** Model selection table ranging the top 10 models assessed for estimating numbers of observations per individual per year in the chick‐rearing period.

AIC	ΔAIC	Model
**13913.94**	**0**	**sex** × **(chick_period + age + herring_size) + seen_before + (1 | birdID) + (1 | year)**
13914.09	0.16	sex + chick_period + age + herring size + seen_before + (1 | birdID) + (1 | year) + sex:chick_period + sex:age
13920.71	6.78	sex + chick_period + age + herring_size + seen_before + (1 | birdID) + (1 | year) + sex:chick_period + sex:herring_size
13921.76	7.82	sex + chick_period + age + herring_size + seen_before + (1 | birdID) + (1 | year) + sex:chick_period
13984.51	70.57	sex + chick_period + age + herring_size + seen_before + (1 | birdID) + (1 | year) + sex:age + sex:herring_size
13985.63	71.70	sex + chick_period + age + herring_size + seen_before + (1 | birdID) + (1 | year) + sex:herring_size
13992.42	78.48	sex + age + herring_size + seen_before + (1 | birdID) + (1 | year)
13994.09	80.15	sex + chick_period + age + seen_before + (1 | birdID) + (1 | year)
13994.40	80.46	sex + chick_period + age + herring_size + seen_before + (1 | birdID) + (1 | year)
13994.92	80.98	sex + chick‐period + age + herring_size + seen_before + (1 | birdID) + (1 | year) + sex:age

*Note*: Preferred model marked in bold. All models have observation effort (hours) as an offset term.

## 4 DISCUSSION

Our study documents a series of sex‐ and age‐dependent responses of Atlantic puffins to changes in breeding conditions, as indicated by two different proxies; the average length of the chick period – an indicator of the growth and survival of the nestling (e.g., Durant et al., 2006) – and the quality of the key prey, age 0 herring, it is fed by the parents (e.g., Durant et al., 2003; Figure 2). These results also indicates that the sex ratio of birds sitting out on the colony to some extent reflects both the current conditions for bringing up the chicks and how long the season has progressed. From a monitoring perspective, this may prove useful as an additional source of information to assess breeding success when such information is otherwise difficult to obtain, e.g., because of limitations in field‐work efforts. Besides some inconclusive studies summarised by Harris (1986) and the study of Creelman and Storey (1991), showing that females incubate more and feed the chick more often than the males, which engage more in nest defence, there is little published evidence on sexual differences in activity budgets for puffins. Fitzsimmons (2018) did however show that females not only fed the chick more often than the males, but that they also increased their feeding effort in a year with poorer availability of the principal prey, while the males continued feeding at the same rate as in three better years.

In common guillemots and razorbills *Alca torda*, two other alcid species, parents share incubation and brooding equally and although the female of those species feeds the young more often, the male spends more time at the nest site (Wanless & Harris, 1986). Very similar patterns of parental investment have also been documented in Brünnich's guillemots *Uria lomvia* (Paredes et al., 2006). In contrast to the chick of puffins, however, which is independent from the minute it fledges (e.g., Harris & Wanless, 2011), the chick of these three larger auk species leaves the nest when close to 3 weeks old and is thereafter accompanied and fed at sea by its father for up to 10 weeks (Nettleship & Birkhead, 1985; Varoujean et al., 1979). Consequently, in razorbills and *Uria* guillemots the male overall invests significantly more time caring for the young than do the female, whereas our results indicate the opposite is the case in puffins.

Overall, the response in attendance patterns of male and female puffins to different conditions for breeding corresponded well with our predictions based on sexual differences in life‐history trade‐offs between investment in breeding and own survival. Following our first prediction of “*Different roles*”, the results confirm that males more frequently sit out in the open on the colony than females (Figure 2a1,b1), which suggests they spend more time defending the burrow entrance, perhaps also the nearby habitat structures best suited for resting, preening, watch‐out and easy take‐off. This corroborates earlier findings showing that male puffins spend more time maintaining and defending the nest burrow than females, whereas females incubate longer and feed the offspring more often than males (Creelman & Storey, 1991; Fitzsimmons, 2018). We have no direct observation data to document what the females were doing when not sitting out in the colony, but among 1520 captures of adult puffins controlled and sexed by head + bill measurements when bringing food to their chick in this colony between 1992 and 2019, there were significantly more females (62.5%) than males (37.5%) (950:570, χ^2^ = 94.5, df = 1, *p* < .001; Anker‐Nilssen, unpubl. data).

Based on our second prediction, “*Stay or go*”, we expected that both sexes would be observed less frequently in the poorer years because they should invest less time in reproductive effort and colony attendance when conditions are insufficient to sustain successful breeding. We found support for this prediction as an increase in herring size, which is strongly correlated with fledging success (Durant et al., 2003), led to an increase in observations for both sexes during chick rearing. Observations of males also increased with increasing length of the chick period, whereas those of females decreased markedly. This sharp contrast may at first seem counterintuitive, as both herring size and the length of the chick period are strongly and positively correlated with fledging success (Durant et al., 2003, 2006). However, in many of the years when herring is too small for sustaining chicks sufficiently, the puffins at Hernyken try raising chicks on alternative prey such as age 0 haddock *Melanogrammus aeglefinus* or other, non‐schooling fish prey (Anker‐Nilssen & Aarvak, 2006). As these species are less abundant, the chick‐feeding puffins probably need to spend more time foraging to sustain the chick than in the good herring years, giving them less time to sit out in the colony. The opposite responses of males and females to the length of the chick period thus indicate the females take most of that extra burden, whereas the males seem to reduce their direct parental care the older the chick gets. Possibly, the relationship between the number of observations and the length of the chick period could be non‐linear as demonstrated for breeding success, which peaks around the mean age of fledglings in the best production years (38–44 days; Durant et al., 2006; Harris & Wanless, 2011). We have tested a few non‐linear functions, but these did not improve the model fit, perhaps because the three longest chick periods registered in this colony (1984, 1985 and 1988) were all in years before colour ringing started and thus not covered by our current data set. We therefore chose not to explore this issue further in the current study. Due to our opportunistic sampling of data, we also refrained from testing if males and females visit the colony at different times of day, as documented in some other auk species (e.g., Elliot et al., 2010; Huffeldt et al., 2021; Thaxter et al., 2009).

We did not find full support for our third prediction of “*Unequal levels of investment*”, from which we expected that the two sexes adjust attendance differently in response to changes in conditions for breeding. Contrary to our predictions, both sexes increased their attendance similarly when herring size improved (Figure 2b3). This is a bit surprising, given the increasing difference in attendance with longer chick periods (Figure 2b4) but, most likely, also factors other than prey quality (e.g., prey availability and diversity) influence breeding conditions. It is, however not possible with our current approach to document the factual reason(s) for this difference.

Finally, we document that the difference between male and female attendance increases the longer it takes to provide for the chick (Figure 2b4). This fits our fourth prediction of “*Accumulated level of investment*” and strongly suggests that females are increasingly more willing than males to invest in provisioning for the chick the longer the chick needs such care. According to basic principles of life‐history theory (e.g., Stearns, 1992), this makes sense given that female puffins from the start have invested more in the offspring by producing the egg. Interestingly, the putative age of the birds (time since first capture as an adult) also contributed to the best models, suggesting reproductive experience and investment in previous years also contribute to determine the birds' willingness to uphold their current parental efforts. Apparently, this effect was strongest in females, as could be expected if their life‐history trade‐off is more focussed on fecundity than that of males, which could also explain the marginal tendency for lower survival in females than males (Landsem et al., 2023).

A meta‐analysis of documented trade‐offs between parental effort and survival in birds indicates that females in general are less likely to reduce their chances of survival when increasing their efforts in current reproduction than are males (Santos & Nakagawa, 2012). It also showed that males with increased parental effort suffer a significant reduction in the probability of survival until the next breeding season. Although the reasons for this are poorly understood, the results of a sexual difference in willingness to or cost–benefit of investing in direct care for the offspring should be more pronounced the longer the period of such investments. This is precisely what our results appear to indicate (Figure 2b). In addition, we found a decrease in number of observations with age and observations of females decreased more than those of males. These results could imply that puffins invest more in rearing offspring with age or, alternatively, that young breeders engage more in social interactions in the colony. Age‐specific patterns in reproductive effort have not been studied in Atlantic puffins, but a decline in age‐specific survival (actuarial senescence) has been documented for puffins at Hernyken and two other colonies (Landsem et al., 2023). Whether this decline is due to an increased tendency with age for skipping breeding or investing more in raising offspring, is unknown. A more detailed study on reproductive success and age in puffins would be needed to find the cause(s) for the apparent decline in observations with age for both sexes.

## 5 CONCLUSIONS

The main finding of our study is the clear difference between male and female puffins in their behavioural response during chick rearing to changes in conditions for breeding. The importance of exploring such effects in long‐lived species is highlighted by the increased value of productivity for population viability in areas where conditions for breeding have remained poor over many years (Layton‐Matthews et al., 2023). As discussed above, this is also highly relevant for understanding the dynamics of individual life‐history trade‐offs between investing in reproduction or securing future survival, which may have substantial consequences for the resilience of seabird populations to withstand longer periods of adverse conditions for breeding, an expected consequence of climate change in many marine ecosystems (IPCC, 2022).

## AUTHOR CONTRIBUTIONS

**Tycho Anker‐Nilssen:** Conceptualization (equal); data curation (lead); formal analysis (supporting); funding acquisition (lead); investigation (lead); methodology (lead); project administration (lead); resources (lead); validation (equal); visualization (equal); writing – original draft (lead); writing – review and editing (lead). **Martina Kadin:** Conceptualization (equal); formal analysis (supporting); investigation (supporting); methodology (supporting); validation (supporting); visualization (supporting); writing – original draft (supporting); writing – review and editing (supporting). **Christoffer Høyvik Hilde:** Conceptualization (supporting); formal analysis (lead); investigation (equal); methodology (equal); validation (equal); visualization (lead); writing – original draft (supporting); writing – review and editing (supporting).

## FUNDING INFORMATION

Norwegian Research Council, Grant Number 192141.

## CONFLICT OF INTEREST STATEMENT

None declared.

## Data Availability

All data and R coding used in the analysis are available from the Dryad Digital Repository https://datadryad.org/stash/share/pIhtZU07K481jOb1R8NHhE5UtJTpDbacnPglUXB7drw.
